# Intraosseous anesthesia with solution injection controlled by a computerized system versus conventional oral anesthesia: A preliminary study

**DOI:** 10.4317/medoral.17543

**Published:** 2011-12-06

**Authors:** Rut Beneito-Brotons, David Peñarrocha-Oltra, Javier Ata-Ali, María Peñarrocha

**Affiliations:** 1 DDS, Master in Endodontics. Private practice in Valencia; 2 DDS, Resident of the Master in Oral Surgery and Implantology. Valencia University Medical and Dental School; 3 DDS, Primary Public Health Service Dentist. Valencian Health Service. Master in Oral Surgery and Medicine. Master in Oral Surgery and Implantology. Valencia University Medical and Dental School; 4 Associate Professor of Oral Surgery. Valencia University Medical and Dental School. Valencia, Spain

## Abstract

Objective: To compare a computerized intraosseous anesthesia system with the conventional oral anesthesia techniques, and analyze the latency and duration of the anesthetic effect and patient preference. Design: A simple-blind prospective study was made between March 2007 and May 2008. Each patient was subjected to two anesthetic techniques: conventional and intraosseous using the Quicksleeper® system (DHT, Cholet, France). A split-mouth design was adopted in which each patient underwent treatment of a tooth with one of the techniques, and treatment of the homologous contralateral tooth with the other technique. The treatments consisted of restorations, endodontic procedures and simple extractions. Results: The study series comprised 12 females and 18 males with a mean age of 36.8 years. The 30 subjects underwent a total of 60 anesthetic procedures. Intraosseous and conventional oral anesthesia caused discomfort during administration in 46.3% and 32.1% of the patients, respectively. The latency was 7.1±2.23 minutes for the conventional technique and 0.48±0.32 for intraosseous anesthesia – the difference being statistically significant. The depth of the anesthetic effect was sufficient to allow the patients to tolerate the dental treatments. The duration of the anesthetic effect in soft tissues was 199.3 minutes with the conventional technique versus only 1.6 minutes with intraosseous anesthesia – the difference between the two techniques being statistically significant. Most of the patients (69.7%) preferred intraosseous anesthesia. Conclusions: The described intraosseous anesthetic system is effective, with a much shorter latency than the conventional technique, sufficient duration of anesthesia to perform the required dental treatments, and with a much lesser soft tissue anesthetic effect. Most of the patients preferred intraosseous anesthesia.

** Key words:**Anesthesia, intraosseous, oral anesthesia, infiltrating, mandibular block, Quicksleeper®.

## Introduction

Intraosseous injection may be an alternative to conventional oral anesthesia, allowing direct injection of the local anesthetic into the cancellous bone adjacent to the target tooth, followed by rapid diffusion and an almost immediate effect (1,2).

Four systems currently allow clinical application of this technique: Stabident® (Fairfax Dental, Miami, USA)(3-5), X-Tip® (X-Tip Technologies, Lakewood, NJ, USA)(3,6), Intraflow® (IntraVantage, Plymouth, MN, USA)(2), and the more recently introduced Quicksleeper® (DHT, Cholet, France)(1,7), which has been used in the present study. This computerized system deposits the anesthetic solution in the cancellous bone of the tooth to be treated, after perforating the cortical layer in a single step and without having to change needle, thanks to the use of instrumentation specifically designed for this purpose.

The present study compares the mentioned computerized intraosseous anesthesia system with conventional oral anesthesia, and analyzes the latency and duration of the anesthetic effect and patient preference.

## Material and Methods

A simple-blind prospective study was made between March 2007 and May 2008. Each patient was subjected to two anesthetic techniques: conventional and intraosseous using the Quicksleeper® system (DHT, Cholet, France). Anesthesia was always carried out by the same person (the first author). A split-mouth design was adopted in which each patient underwent treatment of a tooth with one of the techniques, and treatment of the homologous contralateral tooth with the other technique. A 7-day interval was allowed between one procedure and the other. The patient was unaware of which of the two techniques would be used first, and moreover the order of the interventions was altered.

The dental treatments consisted of reconstructions with composite, root canal treatments of teeth with vital pulp tissue, and simple extractions. To this effect we selected patients with symmetrical dental disorders requiring the same treatments.

The following inclusion criteria were established: 1) patients between 18-65 years of age; 2) absence of disease antecedents (diabetes mellitus, heart disease, arterial hypertension; 3) no analgesic drug use; 4) no history of oral or soft tissue infections; and 5) preserved pulp vitality as determined by thermal and electrical methods. Patients with periodontal (periodontal pockets or dental mobility) or radiological alterations (bone loss or periapical radiotransparencies) were excluded from the study.

A previously established protocol was applied to all patients, followed by coding for statistical analysis. The patients were instructed to score their discomfort during the administration of anesthesia by means of a verbal pain scale comprising four descriptions of pain (from no pain to intense pain). In the case of conventional anesthesia, the latency of anesthetic effect was evaluated measuring the time from completion of the anesthetic maneuver until the patient reported soft tissue numbness. The treatment procedure was started at this point, and if the patient experienced discomfort, we waited for the numbness sensation to increase – with due registry of the additional time. In the case of intraosseous anesthesia, the intervention was started immediately after the injection. Measurement of the injection effect was also measured immediately after completing the anesthetic maneuver.

The duration of the numbness sensation was measured by instructing the patients to record the duration of the anesthetic sensation in minutes. This information was supplied by the patient on occasion of the following visit one week later in which the second treatment procedure was carried out. In the case of the intraosseous technique, the duration was recorded on the day of the intervention.

Lastly, the patients were asked to specify their preference for one technique or the other.

All patients gave written informed consent for inclusion in the study, which was carried out in abidance with the principles of the Declaration of Helsinki and with due authorization from the local Ethics Committee.

**Figure 1:**
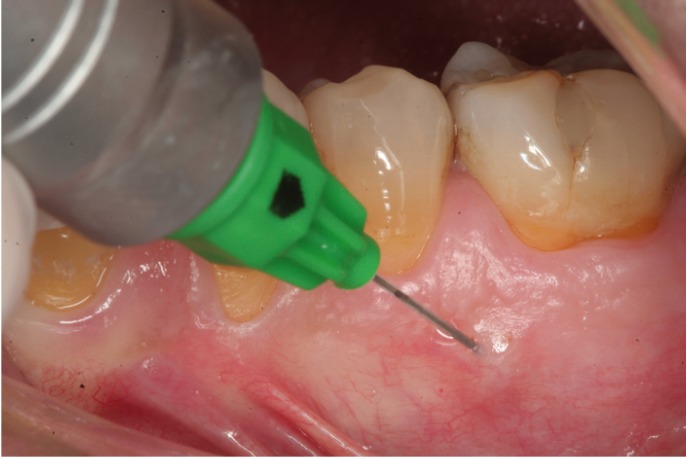
Mucosal anesthesia before cortical perforation.

**Figure 2:**
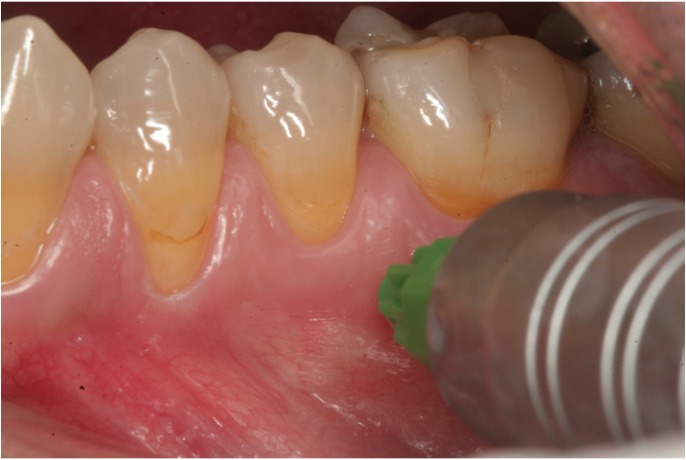
Perforation of the cortical layer, reaching the cancellous bone.

**Figure 3:**
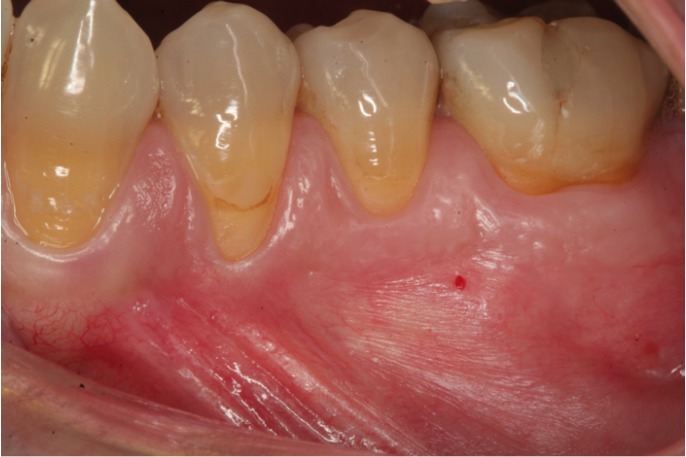
Minimal mucosal damage is observed after perforation of the cortical layer.

Conventional anesthesia (truncal and infiltrating anesthesia) was carried out using the Aspiject® syringe (Laboratorios Inibsa, Barcelona, Spain) with an auto-aspiration system and a 25-mm needle. Intraosseous anesthesia in turn was administered with the Quicksleeper® system, following the instructions of the manufacturer (Figs. 1,2,3). This system consists of an electronic control unit which determines the injection and rotation parameters), a dual pedal (for starting injection or rotation), and a manual device housing the motors for injection and rotation. Use was made of the Trancrt-S® needle, which has two asymmetrical bevels designed to ensure painless penetration. The needle measures 0.4 mm in diameter and 12 mm in length.

The anesthetic solution used with both techniques was 4% articaine with adrenalin 1:100,000 (Laboratorios Inibsa®, Barcelona, Spain).

The SPSS version 12 statistical package for Microsoft Windows was used for the statistical analysis –significance with the different tests being considered for p ≤ 0.05. The Wilcoxon test was used to assess patient discomfort with the anesthetic techniques, while the Student t-test was used to analyze latency and the duration of soft tissue anesthetic effect. The McNemar test in turn was applied for assessing the intensity of effect, and a binomial test was used to explore the patient preferences.

## Results

The study series comprised 12 females and 18 males with a mean age of 36.8 years (range 18-65). The 30 subjects underwent a total of 60 anesthetic procedures.

Intraosseous and conventional oral anesthesia caused discomfort during administration in 46.3% and 32.1% of the patients, respectively ( Table 1). Although reported discomfort was greater with intraosseous anesthesia, the differences were not statistically significant.

**Table 1:**
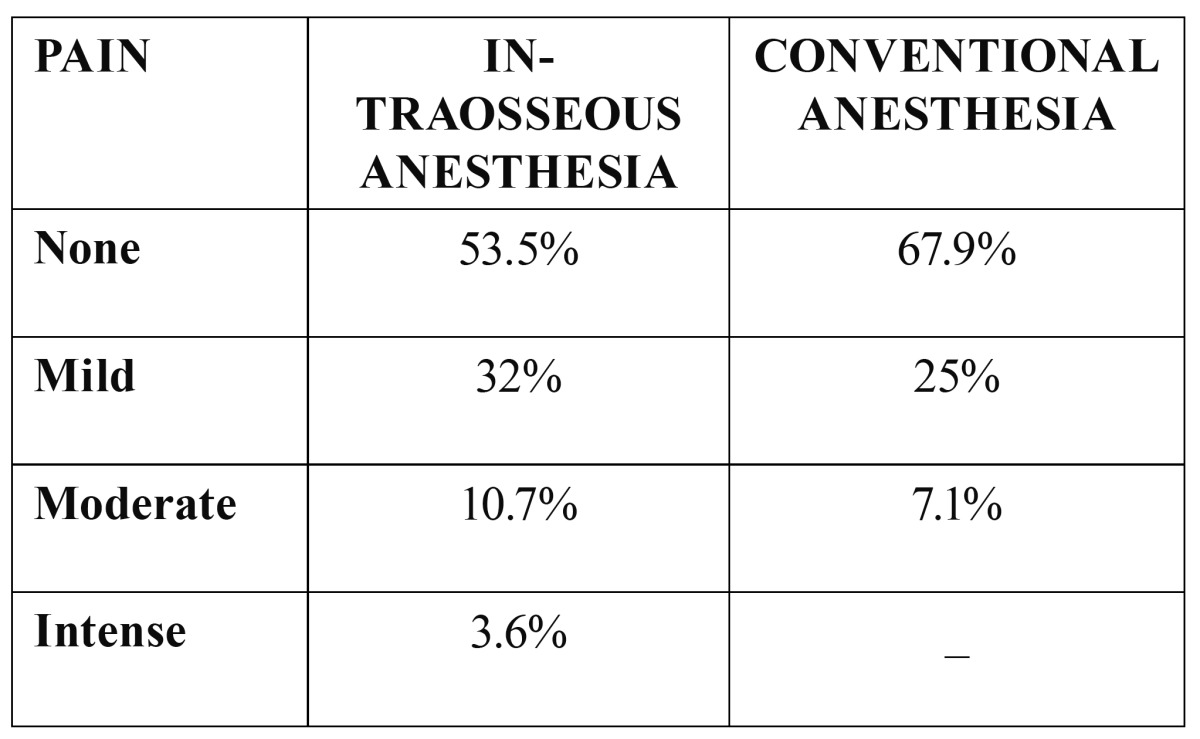
Sensation during injection.

The same dental treatments were provided in both groups. The depth of the anesthetic effect was sufficient with both techniques to allow the patients to tolerate the dental treatments, including endodontic treatment of vital teeth and simple extractions.

The latency was 7.1±2.23 minutes (range 3-14) with the conventional technique, and 0.48±0.32 minutes (range 0-4) with intraos-seous anesthesia – the difference being statistically significant (p<0.05).

The duration of the anesthetic effect in soft tissues was 199.3 minutes with the conventional technique versus only 1.6 minutes with intraosseous anesthesia – the difference between the two techniques being statistically significant (p<0.05).

Most of the patients (69.7%) preferred intraosseous anesthesia, while 23.3% preferred the conventional technique – the difference between the two being statistically significant (p<0.05).

## Discussion

The Quicksleeper® (1) combines electronic rotation of the needle for penetrating the bone with an electronic anesthetic solution release system. As a result of the electronic control, this intraosseous anesthesia technique avoids tissue heating during perforation and ensures slow injection of the anesthetic solution – thus theoretically resulting in less patient discomfort. The discomfort rate associated with this system varies between 11-82% (6,8,9), depending on the clinical study and methodological design involved. In our series, discomfort with the Quicksleeper® was reported by 46.3% of the subjects.

Quarnstrom (10), in a comparative study of latency, found inferior dental nerve block to take 7 minutes, while effective anesthesia was achieved in only 36 seconds with the intraosseous anesthesia technique. In the study published by Leonard (11), latency with the conventional technique was 8-17 minutes, versus 10-120 seconds with intraosseous anesthesia. Our own findings were similar, with latency values of 7.1±2.23 minutes for the conventional technique and 0.48±0.32 for intraosseous anesthesia.

The depth of the anesthetic effect was sufficient with both techniques to allow the patients to tolerate the dental treatments, including endodontic treatment of vital teeth and simple extractions. In a study involving 30 patients subjected to conventional anesthesia with 4% articaine (12), the duration of the anesthetic effect was 220.8 minutes, versus 199.3 minutes in our own study (compared with only 1.6 minutes in the case of intraosseous anesthesia).

In a study (1) that likewise made use of the Quicksleeper® system, 58.9% of the 50 included patients claimed to prefer intraosseous anesthesia to the conventional technique. In our series the proportion of patients preferring intraosseous anesthesia was slightly higher (69.7%).

The described intraosseous anesthetic system is effective, with a much shorter latency than the conventional technique, sufficient duration of anesthesia to perform the required dental treatments, and with a much lesser soft tissue anesthetic effect. Most of the patients preferred intraosseous anesthesia.
